# Supplementary figures and images for: A targeted CRISPR-Cas9 mediated F0 screen identifies genes involved in establishment of the enteric nervous system
Source: PLoS One. 2024 May 29;19(5):e0303914. doi: 10.1371/journal.pone.0303914 (PMC11135701; doi:10.1371/journal.pone.0303914)

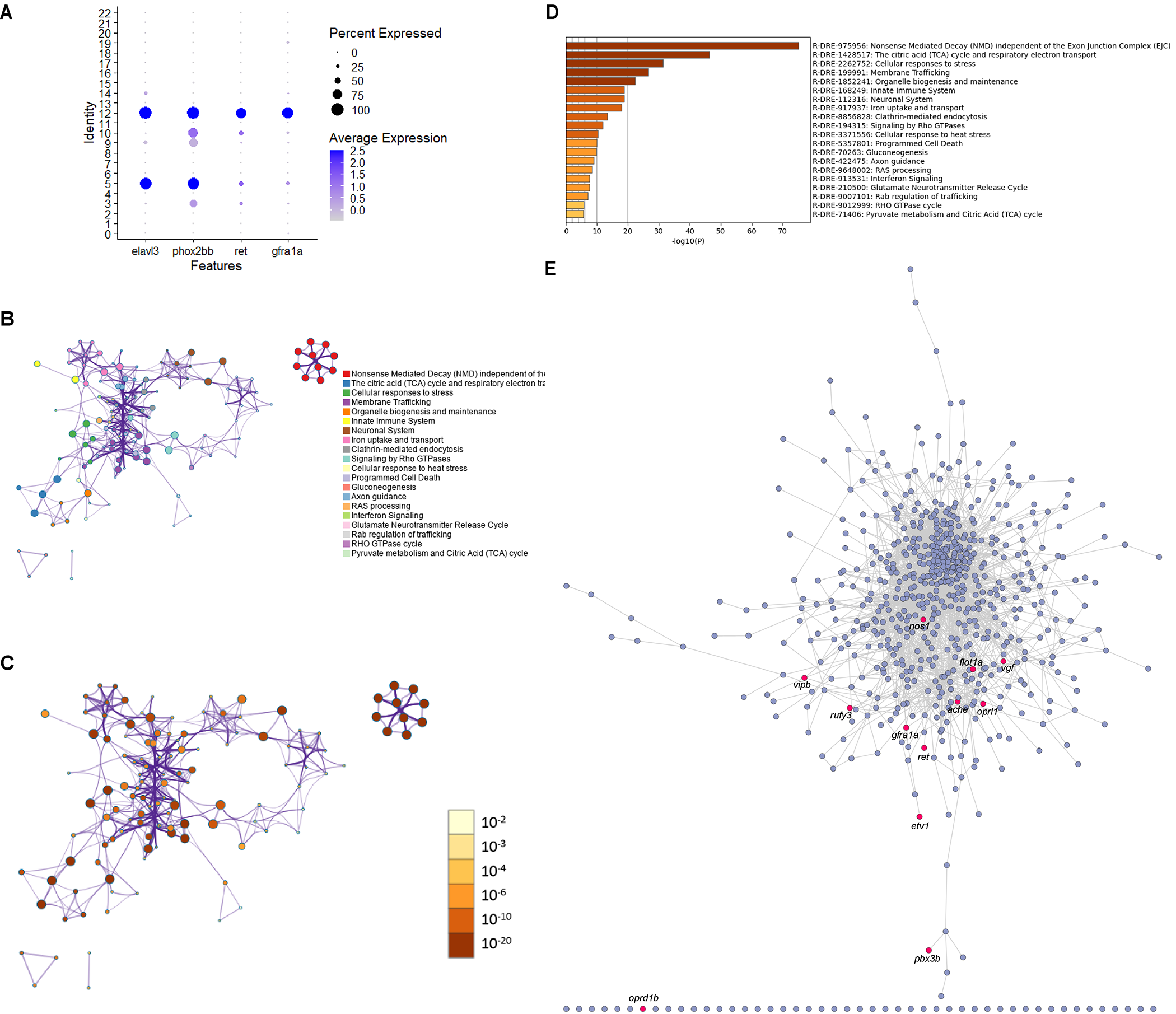

Supplement: S1 Fig — (A) Dot plot depicts the expression level of enteric neuron-specific markers across individual clusters generated within the main 68–70 hpf tSNE sox10:GFP+ dataset [20]. Clusters 5 and 12 prominently expressed enteric neuron markers. Dot size depicts the cell percentage for each marker, and the color summarizes the average expression levels for each gene. (B, C) Metascape network of enriched terms from the Reactome zebrafish gene set, colored by cluster (B) or by p-values (C), this network was based on the differentially expressed genes table from Seurat scRNA seq analysis of sub-cluster 3 (S1 Data). (D) Bar graph of top 20 enriched Reactome zebrafish terms across input gene lists colored by p-values. (E) STRING network based on the complete sub-cluster 3 depicting the selected genes for the CRISPR screen in red. (TIF) [file pone.0303914.s001.tif]

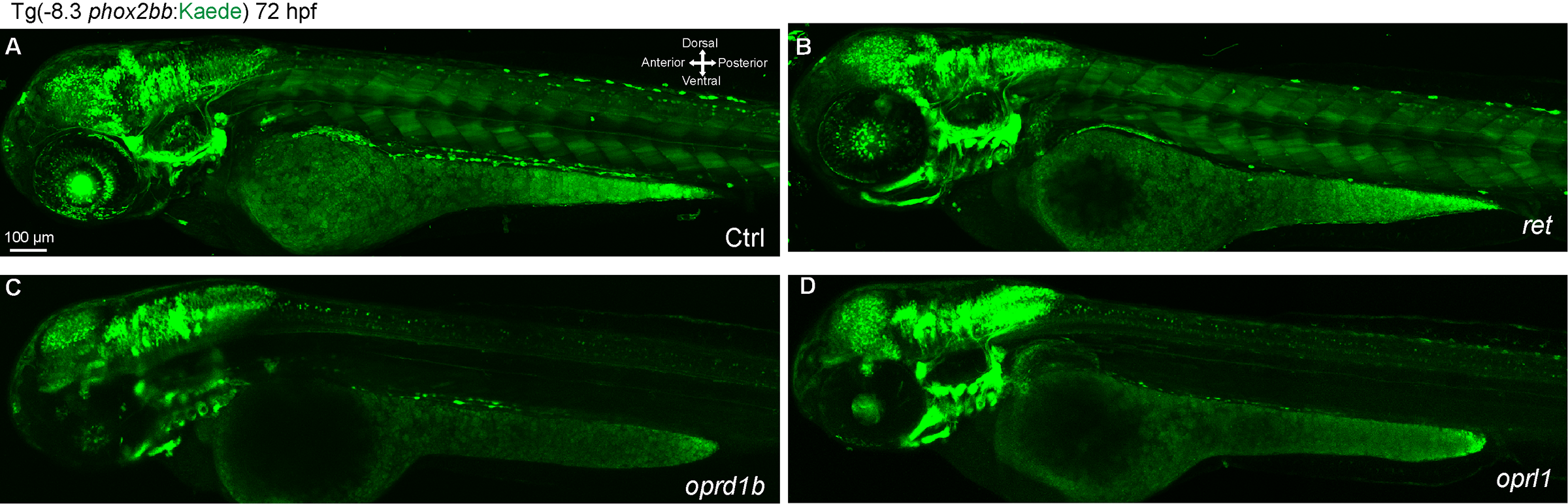

Supplement: S2 Fig — Confocal images of whole Tg(-8.3phox2bb:Kaede) crispants for ret (B), oprd1b (C), oprl1 (D), and control (A) at the 72 hpf, with no visible drastic effects (≥ 3 experiments with 3 biological replicates). (TIF) [file pone.0303914.s002.tif]

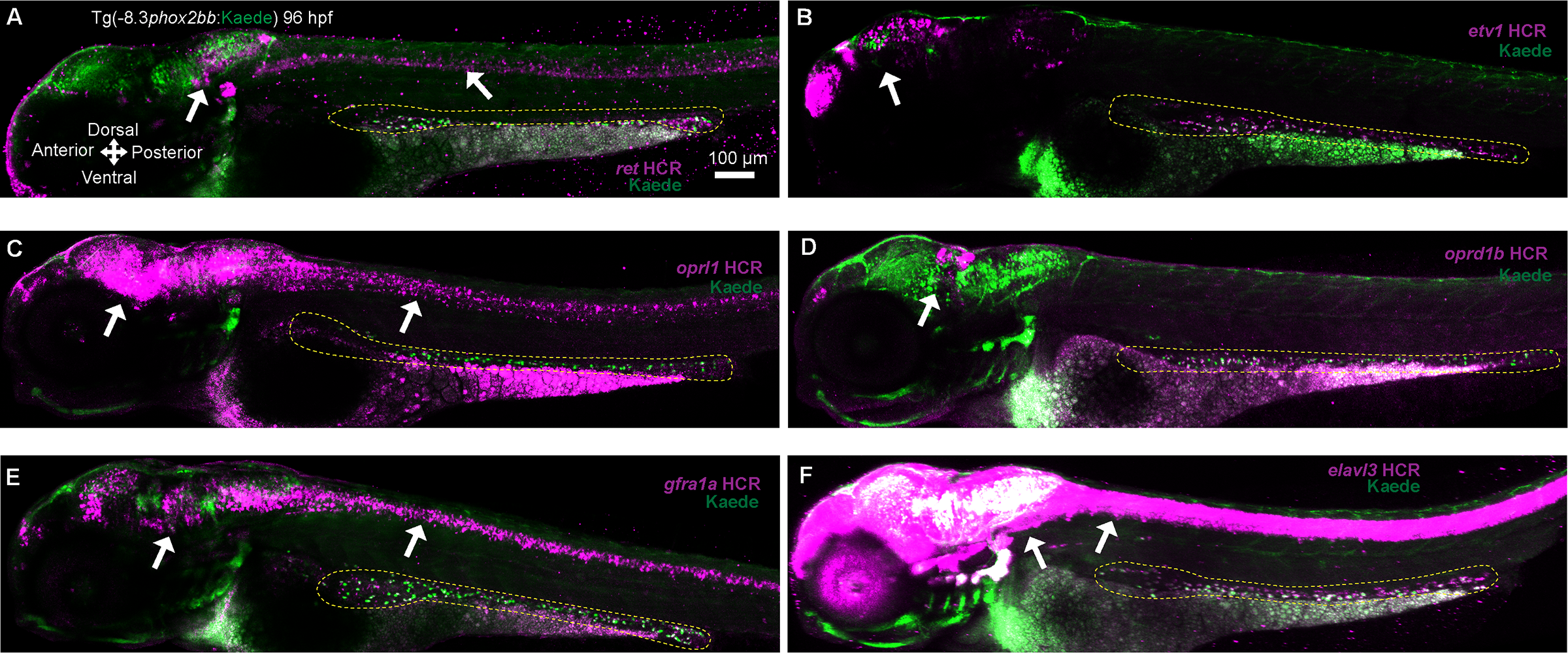

Supplement: S3 Fig — Confocal images show HCR-assayed expression of ret (A), etv1 (B), oprl1 (C), oprd1b (D), gfra1a (E), and elavl3 (F) in whole Tg(-8.3phox2bb:Kaede) larvae at the 96 hpf, revealing expression along the spinal cord and brain regions. The Kaede signal is green, and the specific mRNA signal is magenta. White arrows depict the signals of the probes in the brain or spinal cord. Dashed yellow lines surround the ENS. (TIF) [file pone.0303914.s003.tif]
